# Embryo development in association with asymbiotic seed germination *in vitro* of *Paphiopedilum armeniacum* S. C. Chen et F. Y. Liu

**DOI:** 10.1038/srep16356

**Published:** 2015-11-12

**Authors:** Yan-Yan Zhang, Kun-Lin Wu, Jian-Xia Zhang, Ru-Fang Deng, Jun Duan, Jaime A. Teixeira da Silva, Wei-Chang Huang, Song-Jun Zeng

**Affiliations:** 1 Key Laboratory of South China Agricultural Plant Molecular Analysis and Gene Improvement, South China Botanical Garden, Chinese Academy of Sciences, Guangzhou 510650, China; 2 University of Chinese Academy of Sciences, Beijing 100049, China; 3 P. O. Box 7, Miki-cho post office, Ikenobe 3011-2, Kagawa-ken, 761-0799, Japan; 4 Shanghai Chen Shan Botanical Garden, Shanghai 201602, China

## Abstract

This paper documents the key anatomical features during the development of *P. armeniacum* zygotic embryos and their ability to germinate asymbiotically *in vitro*. This study also examines the effect of media and seed pretreatments on seed germination and subsequent seedling growth. Seeds collected from pods 45 days after pollination (DAP) did not germinate while 95 DAP seeds displayed the highest seed germination percentage (96.2%). Most seedlings (50%) developed to stage 5 from 110 DAP seeds whose compact testa had not yet fully formed. Suspensor cells were vacuolated, which enabled the functional uptake of nutrients. The optimum basal medium for seed germination and subsequent protocorm development was eighth-strength Murashige and Skoog (1/8MS) for 95 DAP seeds and ¼MS for 110 DAP seeds. Poor germination was displayed by 140 DAP seeds with a compact testa. Pretreatment of dry mature seeds (180 DAP) with 1.0% sodium hypochlorite solution for 90 min or 40 kHz of ultrasound for 8 min improved germination percentage from 0 to 29.2% or to 19.7%, respectively. Plantlets that were at least 5 cm in height were transplanted to a Zhijing stone substrate for orchids, and 85.3% of plantlets survived 180 days after transplanting.

*Paphiopedilu*m is a rare orchid genus, which is comprised of about 96–100 species around the world^1^,^2^,^3^. The rarity, beauty and value of *Paphiopedilum* species, popularly known as slipper orchids, have always captured the interest of orchid growers and hobbyists. Wild populations of *Paphiopedilum* are under the constant threat of extinction due to overcollection for use as ornamental plants or as breeding parents. This loss of suitable habitats is caused exclusively by anthropogenic activity in response to the trade of this orchid. Incidentally, the trade of all *Paphiopedilum* species, which are listed in the Convention on International Trade in Endangered Species of Wild Fauna and Flora (CITES) Appendix I, is prohibited^4^,^5^,^6^. *P. armeniacum* S. C. Chen et F. Y. Liu is one of the most fascinating *Paphiopedilum* species because of its characteristic golden flowers. Currently (last assessed on September 6, 2015), 124 hybrids use *P. armeniacum* as the parent, 39 as seed parents and 85 as pollen parents^7^. *P. armeniacum* is a terrestrial or lithophytic orchid that is only distributed in the western part of Yunnan province, China, along the Nu River from Shidian county in the south to the North of Fugong county and the Southwest of Weixi county in the North, and flowers from March to May in the wild^2^,^8^ (Fig. 1). *P. armeniacum* is currently listed as endangered in The IUCN Red List of Threatened Species, version 2014.3. (www.iucnredlist.org, 2015).

The success of tissue culture protocols based on *ex vitro*-derived *Paphiopedilum* explants is limited due to the rarity of materials, difficulties caused by bacterial and fungal decontamination and the poor development of explants that survive under *in vitro* conditions^6^,^9^,^10^. Asymbiotic seed germination is an efficient method for the large-scale propagation of orchids^11^. Several protocols for *in vitro* seed germination of *Paphiopedilum* species, including *P. armeniacum*, have been described^6^. The development of *Paphiopedilum* zygotic embryos during seed germination and protocorm development *in vitro* is stimulated by suitable pretreatment, including with sodium hypochlorite (NaOCl) or ultrasound^5^,^12^,^13^,^14^. *P. armeniacum* seed germination and subsequent protocorm development are significantly influenced by capsule maturity, seed pretreatment, medium composition, culture conditions, culture method, as well as other conditions^6^,^13^,^14^,^15^,^16^,^17^. In addition, asymbiotic seed germination percentage is subtantially lower (0–60%) than in other *Paphiopedilum* species while some experimental results, including seed germination percentage on the same medium, are inconsistent^13^,^14^. There are some reports on zygotic embryo development in *Paphiopedilum* species^18^,^19^,^20^,^21^,^22^. However, no analysis exists of the comparison between embryo/seed morphological characteristics, seed germination percentage and protocorm development. Moreover, no studies to date have used a pretreatment with NaOCl or ultrasound to facilitate seed germination and protocorm development of *P. armeniacum*.

Based on this background, there were three primary goals of this study. Firstly, to investigate the key anatomical features during zygotic embryo development of *P. armeniacum* in association with the ability of zygotic embryos to germinate asymbiotically *in vitro*. Secondly, to examine the effect of culture media and seed pretreatments on seed germination and subsequent seedling growth, with the ultimate purpose of increasing both. Thirdly, to establish an effective *in vitro* propagation system for the large-scale propagation of *P. armeniacum* to meet commercial needs and to eventually reestablish populations of this threatened orchid species back into the wild.

## Results

### Embryo development

**Figure 2:**
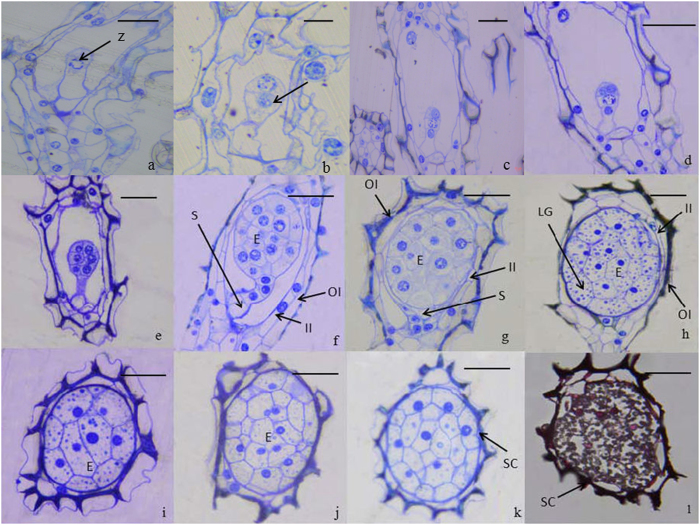
Histological study of embryo development in *P. armeniacum*. (**a**) Zygote (Z), soon after fertilization at 45 days after pollination (DAP); (**b**) two-cell pre-embryo (arrowhead) at 52 DAP; (**c**) three-cell pre-embryo at 59 DAP; (**d**) T-shaped pre-embryo with four cells at 66 DAP; (**e**) six-cell pre-embryo at 73 DAP; (**f**) early globular embryo with a curving suspensor (S) at 80 DAP; (**g**) globular embryo at 87 DAP, the outer layer cells of the outer integument begin to dehydrate and the suspensor degenerates; (**h**) globular embryo at 94 DAP, the inner layer cells of the outer integument begin to dehydrate, and starch and lipid globules (LG) accumulate; (**i**) the globular embryo at 101 DAP with a degenerated suspensor; (**j**) the globular embryo is very close to the seed coat (SC) at 115 DAP; (**k**) the embryo has matured with a compact SC and no further morphological changes at 122 DAP; (**l**) the SC is very compact and thick at 180 DAP. E: embryo; II, inner integuments; OI, outer integuments. Scale bar = 50 μm.

The major microscopic structural events taking place in developing capsules of *P. armeniacum* after pollination are described in Table 1 and displayed in Fig. 2. At 45 days after pollination (DAP), about 80% of ovules had been fertilized and zygotic embryos began to develop, becoming elongated (Fig. 2a). The nucleus was localized toward the chalazal end and a prominent vacuole was found at the micropylar end. At 52 DAP the zygote divided into two cells (Fig. 2b) and derivatives of the basal cell gave rise to the suspensor, which only consisted of a single cell that was highly vacuolated. At 59 DAP a three-cell pre-embryo was observed (Fig. 2c). At 66 DAP the cell at the terminus divided anticlinally giving rise to a T-shaped pre-embryo with four cells (Fig. 2d). At 73 DAP the middle two cells divided forming a spherical six-cell pre-embryo (Fig. 2e). At 80 DAP a multi-cell early globular embryo displayed a curving suspensor (Fig. 2f). A globular embryo began to form at 87 DAP when the outer layer of cells of the outer integument began to dehydrate and the suspensor started to degenerate (Fig. 2g). At 94 DAP a globular embryo proper formed, the inner-layer cells of the outer integument began to dehydrate, the suspensor continued to degenerate, and starch and lipid globules accumulated, events that epitomize the storage of nutrients (Fig. 2h). At 101 DAP the globular embryo had fully developed, the suspensor had fully degenerated, and starch and lipid globules continued to accumulate (Fig. 2i). At 108 DAP the inner testa disappeared, and there was additional accumulation of starch and lipid globules. At 115 DAP the cells of the inner integument degenerated and the globular embryo was very close to forming a testa rich in starch and lipid globules (Fig. 2j). By 122 DAP the embryo had fully matured, evidenced by a compact dark testa, and no further morphological changes took place (Fig. 2k). At 140 DAP mature seed had desiccated and at 150 DAP seeds were fully mature. At 220 DAP, the ripe capsule split.

### Effect of the degree of seed maturity on TTC staining and *in vitro* germination

Seed germinated at 120 days after culture. 2,3,5-Triphenyl tetrazolium chloride (TTC) staining percentage was significantly affected by the degree of seed maturity (Table 2). Pre-embryos at 45 DAP did not stain or germinate. There were no significant differences between seed germination and TTC staining percentage in 55 or 65 DAP pre-embryos. TTC staining percentage was significantly higher than seed germination percentage at 75 or 85 DAP. Highest seed germination percentage (96.9%) and TTC staining percentage (95.7%) were observed in 95 DAP seeds. These values were significantly higher than during all other collection periods. However, TTC staining percentage (82.3%) was still significantly higher (66.8%) in 110 DAP seeds. At 120 DAP TTC staining percentage was lower than seed germination percentage in any collection period. Although highest seed germination percentage was observed in 100 DAP seeds, 140 DAP seeds developed faster and some protocorms could develop to stage 4. A correlation (Pearson’s R = 0.684; P = 0.05) was found between TTC embryo staining and seed germination.

At 180 days after culture, 95 DAP seeds showed highest germination percentage (96.2%), which was significantly higher than all other collection periods, but the percentage of protocorm necrosis was also highest (20.0%). Although the next highest seed germination percentage was 75.0% in 110 DAP seeds, a value that was also significantly higher than all other collection periods, 50% of the protocorms developed to stage 5. This value was significantly higher than all other collection periods (Table 3). Therefore, 110 DAP seeds were assumed to be most suitable for protocorm development.

### Effect of basal media on germination *in vitro*

Seeds of 95 or 110 DAP showed a different response to most basal media at 180 days after culture (Table 4 and Table 5). Highest total seed germination percentage of 95 DAP seeds was observed on quarter-strength Murashige and Skoog^23^ (¼MS) and on eighth-strength (⅛MS) basal media relative to the other eight tested basal media. However, ⅛MS was most suitable for subsequent protocorm development. When ⅛MS was used, seed germination percentage at stage 5 was 40.5%, which was significantly higher than the other nine tested basal media, including 30.1% seed germination on ¼MS medium. This latter medium was most suitable for germination of 110 DAP seeds and subsequent protocorm development, resulting in the highest total seed germination percentage (75.0%) with 50% of protocorms developing to stage 5. These values were significantly higher than on the other nine tested basal media. Therefore, ⅛MS medium proved to be the most appropriate basal medium for seed germination and protocorm development of 95 DAP seeds while ¼MS was most suitable for 110 DAP seeds.

### Effect of NaOCl on TTC staining and germination of mature seeds

Microscopic observations showed that the dark testa of *P. armeniacum* seeds became greyish white after treatment with NaOCl. TTC did not stain fully mature (180 DAP) seed, which germinated on ¼MS medium supplemented with 0.5 mg l^−1^ α-naphthaleneacetic acid (NAA), 10% coconut water (CW) and 1.0 g l^−1^ activated charcoal (AC) at 180 days after culture. The transparency of the testa increased as the exposure period to NaOCl was prolonged. TTC staining, seed germination percentage and protocorm development were affected by the concentration and duration of NaOCl treatment. TTC staining and seed germination percentage were highest when 180 DAP seeds were pretreated with NaOCl containing 1.0% available chlorine for 90 min. These values were significantly higher than all other treatments and these processes were accompanied by the development of most protocorms to stages 4 and 5 (Table 6). However, when seeds were pretreated with the lowest NaOCl concentration (0.5% available chlorine) and for the shortest exposure period (30 min), or the highest NaOCl concentration (1.5% available chlorine) and for the longest exposure period (120 min), seed germination percentage (5.7% and 5.5%, respectively) and TTC staining percentage (2.5% and 3.8%, respectively) were significantly lower than all other treatments (Table 6). After NaOCl pretreatment, a strong correlation (Pearson’s *R* = 0.939; *P* = 0.01) was found between TTC embryo staining and seed germination.

### Effect of ultrasonication pretreatment on TTC staining and germination of mature seeds

TTC staining and germination percentage gradually increased when the seeds were ultrasonicated for 2 to 8 min but decreased when ultrasonication exceeded 10 min. However, highest TTC staining (19.7%) and germination percentage (25.4%) were observed after 8 min of ultrasonication, which was accompanied by the simultaneous development of most protocorms to stages 4 and 5 (Table 7). After ultrasonication pretreatment, a strong correlation (Pearson’s *R* = 0.989; *P* = 0.01) was found between TTC embryo staining and seed germination.

### Effect of organic amendments on plantlet growth *in vitro*

**Figure 3:**
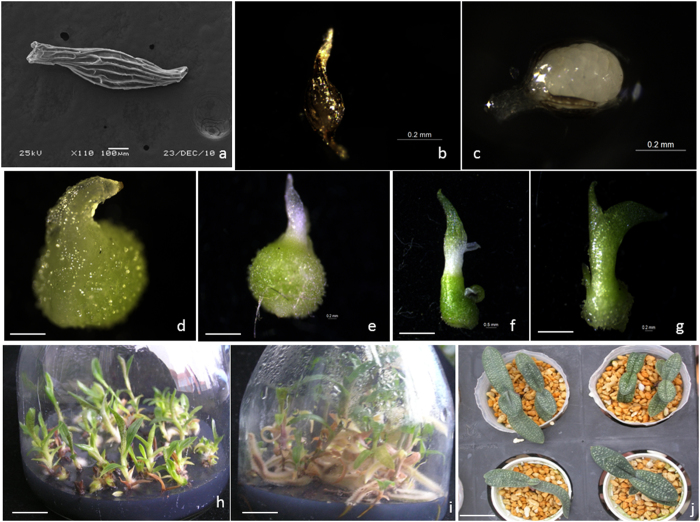
*In vitro* seed germination and seedling development of *P. armeniacum*. (**a**) Dry mature seed of *P. armeniacum* observed by scanning electron microscopy; (**b**) stage 0, swelling seeds, ungerminated; (**c**) stage 1, testa ruptured; (**d**) stage 2, appearance of the shoot; (**e**) stage 3, appearance of the shoot and rhizoids; (**f**) stage 4, emergence and elongation of first leaf; (**g**) stage 5, presence of two or more leaves; (**h**) development of seedlings on ¼MS medium supplemented with 1.0 mg l^−1^ NAA, 10% CW, 1.0 g l^−1^ peptone, and 1.0 g l^−1^ AC; (**i**) seedling growth on Hyponex N026 medium supplemented with 1.0 mg l^−1^ NAA, 1.0 g l^−1^ peptone, 50 g l^−1^ BH, and 1.0 g l^−1^ AC; (**j**) transplanted plantlets after 6 months’ acclimatization in the greenhouse. Scale bars: (**a**) 100 μm, (**b**), (**c**) and (**d**) 0.2 mm, (**e**) 0.5 cm, (**f**) and (**g**) 1.0 cm, (**h**) and (**i**) 1.5 cm, (**j**) 3.0 cm.

The seedlings arising from seed that germinated on ¼MS medium supplemented with 0.5 mg l^−1^ NAA, 10% CW and 1.0 g l^−1^ AC after 180 days were first subcultured on ¼MS medium supplemented with 1.0 mg l^−1^ NAA, 10% CW, 1.0 g l^−1^ peptone, and 1.0 g l^−1^ AC. These seedlings could grow to over 2 cm in height following 90 days of culture (Fig. 3h, data not shown). When plantlets 2 cm in height were transferred to Hyponex N026 medium supplemented with 1.0 g l^−1^ peptone, 1.0 mg l^−1^ NAA, and 1.0 g l^−1^ AC and different types and concentrations of organic amendments, plantlet growth *in vitro* was significantly affected (Table 8). Following the assessment of all growth parameters (mean number of shoots per seedling, height of tallest shoots, number of leaves in tallest shoots, and length and diameter of longest roots), Hyponex N026 medium supplemented with 50 g l^−1^ banana homogenate (BH) was found to be most suitable for plantlet growth *in vitro* since it resulted in a favorable number of shoots, tallest shoots, most leaves and moderate root length and diameter. These are aspects that are appropriate for transplanting and that ensure effective *ex vitro* plantlet growth (Fig. 3i).

### Greenhouse acclimatization

Plantlets grew vigorously 30 days after transplanting. After 90 days of transplanting, the highest percentage of plantlet survival (90.7%) was observed on Chilean sphagnum moss which was significantly higher than on sieved peat, commercial sand for orchids or substrate mixture 2 (Table 9). The lowest plantlet survival percentage (74.3%) was observed on commercial sand for orchids, which was not significantly different to sieved peat or substrate mixture 2. After 180 days of transplanting, the highest plantlet survival percentage (85.3%) was observed on Zhijing stone for orchids (Fig. 3j), which was also significantly higher than on sieved peat, commercial sand for orchids or substrate mixture 2, but the lowest plantlet survival percentage (63.7%) was observed on commercial sand for orchids. This latter substrate performed most poorly among all tested substrates. Relative to plantlet survival percentage on the same substrate at 90 and 180 days after transplanting, both values were not significantly different on Zhijing stone for orchids, substrate mixture 1 or substrate mixture 3; all three substrates included Zhijing stone for orchids (Table 9). Since the roots of transplanted seedlings almost never elongated on Chilean sphagnum moss, Zhijing stone for orchids is most suitable for transplanting seedlings. About 5000 plantlets from all treatments were successfully acclimatized to greenhouse conditions and can be used for ornamental, ecorehabilitation and conservation purposes.

## Discussion

Many reports have indicated that *Paphiopedilum* seed germination *in vitro* is significantly affected by the degree of seed maturity^6^. Asymbiotic seed germination of fully mature *Paphiopedilum* orchid seeds is often difficult and thus immature seeds need to be germinated more readily; moreover, even though mature seeds are suitable for storage, seed germination of mature dry seeds following storage has not yet been reported^5^,^6^,^12^,^13^,^14^,^19^,^21^. There are some reports related to zygotic embryo development in *Paphiopedilum*^18^,^19^,^20^,^21^,^22^. However, analyses between embryo/seed morphological characteristics and seed germination percentage or protocorm development do not exist. Lee *et al.*^21^ reported that the suspensor is the major site of nutrient uptake for the developing zygotic embryo. In the *in vitro* culture of early zygotic embryos, the suspensor may remain the major site of nutrient uptake. These absorbed nutrients are necessary for the further *in vitro* development of the embryo and for protocorm formation and development. In this study, germination percentage of 95 DAP seed was highest, and at this stage, the suspensor had only started to degenerate, making the pathway to active nutrient uptake a realistic option. However, at 95 DAP, the embryo had not yet fully matured and the accumulation of starch and lipid globules was insufficient to sustain subsequent seedling development, resulting in the death of 20% of germinated protocorms. Seed collected from 110 DAP capsules had the next highest seed germination percentage and most seedlings developed to stage 5. In this developmental stage, although the suspensor degenerated and the embryo had fully formed, the compact testa did not form; this state may have permitted the penetration and absorption of water and nutrients^21^. At 120 DAP, seed germination decreased sharply from 66.9% to 37.5% at 110 DAP; at this developmental stage, the embryo was fully mature with a compact and fully formed testa which may have prevented the absorption and permeation of water and nutrients^21^. At 130 DAP, seed germination percentage decreased further, possibly because of additional development of the impermeable testa caused by cutinization and lignification^24^,^25^, the presence of chemical inhibitors such as abscisic acid (ABA), or the lack of certain germination-promoting hormones^26^. Lee found that the endogenous ABA content of *Cypripedium formosanum*, which is also commonly referred to as a slipper orchid, was low at 60 DAP but increased rapidly during 120–150 DAP^27^. A high level of ABA accumulated on the surface wall of the embryo proper and the shrivelled inner integument of mature *C. formosanum* seeds, which coincided with a rapid decrease in seed germination.

Seed germination and seedling development of *Paphiopedilum* is affected by the choice of medium^5^,^6^,^13^. Most *Paphiopedilum* species prefer a low salt medium for seed germination^6^,^13^,^14^,^16^,^28^. However, some experimental results in the literature, such as the germination percentage of seeds of the same species on the same medium, are inconsistent or even contradictory^13^,^14^. Chen *et al.*^13^ reported that Robert Ernst medium (RE) was most suitable for the germination of 120 DAP seed of *P. armeniacum* and P. micranthum and significantly higher than on MS, ½MS, Knudson’s C (KC) and Hyponex media. In contrast, Ding *et al.*^14^ indicated that 1/5MS medium was most suitable for 112 DAP seed germination of *P. armeniacum* and *P. micranthum*, and higher than on RE, ½MS, 1/10MS, KC and Hyponex media. In this study, we found differences in the most appropriate medium for the germination of *P. armeniacum* seed at different stages of seed maturity. The most appropriate medium for younger seeds (95 DAP) was ⅛MS (94.3% seed germination) or ¼MS (96.2% seed germination), but ⅛MS was more suitable for subsequent protocorm development. The percentage of seedlings that developed to stage 5 on ⅛MS was 40.5%, which was significant higher than 30.1% on ¼MS. 110 DAP seeds preferred a higher salt medium (¼MS). This preference may be because younger seeds were sensitive to a higher salt concentration in the medium. This result might also explain why seed germination percentage of some *Paphiopedilum* species is different on the same medium. This is most likely because the collecting period (i.e., age) of seeds is different, and even though the collecting period may be consistent, their maturity may be inconsistent in different growing environments or seed harvest years^29^.

TTC staining is the most widely used biochemical method to evaluate seed viability. Compared to direct germination assays, this method has the advantage of being rapid and suitable for controlled conditions^30^,^31^,^32^. This test has been successfully used with some orchids, including *Cypripedium*^33^,^34^,^35^,^36^. Lanzer *et al.*^33^ reported that the germination percentage of *C. acaule* was always lower than that of embryos stained with TTC. Lee *et al.*^34^ and Zhang *et al.*^35^ obtained the same result for *C. formosanum*. However, Yamazaki and Miyoshi^25^ reported that the mature seeds (140 DAP) of *Cephalanthera falcata* were not stained by TTC. These results are difficult to interpret because mature *C. formosanum* and *C. falcata* seeds have two compact testas, thus cutinization and lignification of the inner integument (often been referred to as the ‘carapace’) is postulated to strengthen the inhibition of embryo growth by mechanical restriction^37^,^38^ or by chemical reactions^25^,^34^,^35^. The seed germination and TTC staining percentage of *Paphiopedilum* SCBG Red Jewel were significantly affected by the degree of seed maturity, but no correlation (Pearson’s *R* = 0.086) was found between TTC embryo staining and seed germination (Zeng *et al.*, unpublished data). In this study, before seed matured, TTC staining percentage was higher than germination percentage but after seed matured, TTC staining of seeds was lower than germination percentage (Table 2). However, in this study, strongly positive correlations (Pearson’s R = 0.939 or 0.989 respectively), were found between TTC embryo staining and seed germination from seeds at different developmental stages or from mature seeds following pretreatment with NaOCl or ultrasonication. These results may reflect differences in the inherent characteristics of different *Paphiopedilum* species or varieties. Nevertheless, mature *Paphiopedilum* seeds have a single testa^19^,^20^,^21^,^22^ and germination percentage is usually higher than *Cypripedium* seeds^6^,^36^, but we found that the TTC staining percentage of *Paphiopedilum* seeds tends to be lower or not stained at all. This difference in TTC staining and seed germination between *Paphiopedilum* and *Cypripedium* could be attributed not only to the permeability of the testa, but may also depend on the reaction of different tissues to TTC dyes^39^,^40^, because the TTC staining assay is based on the activity of seed dehydrogenases^30^.

Mature orchid seeds may have a greater potential for propagation and storage because of a fuller testa and lower water content^22^,^35^. Mature seeds may also assist their survival in harsh climates. Under natural conditions, seasonal changes in temperature, wetting and drying, mechanical abrasion or digestion by mycorrhizal fungi may damage the testa and promote seed germination^34^. However, in this study, 1.3% of fully mature (180 DAP) *P. armeniacum* seeds obtained directly from wet capsules were stained by TTC, while displaying a 4.5% seed germination percentage after culture for 120 days. In contrast, fully mature (180 DAP) dry stored *P. armeniacum* seeds could not be stained by TTC or germinate (data not shown), although germination and protocorm development could be stimulated by suitable pretreatment with NaOCl or ultrasonication, as was also observed in previous reports for orchids, including *Paphiopedilum*^5^,^12^,^33^,^34^,^38^,^41^. The TTC staining of seeds was not studied when they were pretreated with NaOCl or ultrasonication. Pretreatment of NaOCl may erode the testa and disrupt cell wall integrity, thus increasing the permeability of the seed to oxygen and nutrients^24^,^42^,^43^,^44^,^45^, while inhibitors such as ABA may become reduced within seeds^12^,^26^. However, this hypothesis needs to be tested further. The use of ultrasound had been used to increase the germination of mature orchid, possibly because embryos of treated seeds result in cavitation and acoustic microstreaming, two processes that can modify cellular ultrastructure, enzyme stability and cell growth and can also cause breaks in extracellular polymers, release DNA from the nucleus, decrease cell stability, alter cell membrane permeability and modify charges on the surfaces of cells or stimulate protein synthesis in plant cells and protoplasts^46^,^47^. However, in this study, when mature dry seeds (180 DAP) were pretreated with ultrasound, seed germination percentage was higher than TTC staining percentage for all treatments, but when they were pretreated with NaClO containing 1.0% available chlorine for 30, 60 or 90 min or 0.5% available chlorine for 120 min, TTC staining percentage was higher than seed germination percentage. Conversely, in other treatments, an opposite result was observed, and TTC staining percentage of seed treated with ultrasound was lower than with NaClO pretreatment (Table 6; Table 7). This may be caused by some inhibitors that prevent staining in the testa but that can be eliminated by suitable NaOCl pretreatment.

Orchid seed germination and protocorm development are stimulated by organic amendments^5^,^11^,^48^,^49^,^50^,^51^,^52^. Organic amendments include, among others, CW, carrot homogenate (CH), BH, potato homogenate (PH), tryptone or peptone^5^,^6^ but only CH, BH and PH are used for *Paphiopedilum* seedling growth and rooting^6^. Therefore, short, 2-cm tall seedlings were initially subcultured on ¼MS medium supplemented with 1.0 mg l^−1^ NAA, 10% CW, 1.0 g l^−1^ peptone, and 1.0 g l^−1^ AC without CH, BH, PH. In all culture stages, CW can facilitate seed germination and protocorm development possibly because CW contains many different types of biochemicals, including amino acids, vitamins, sugar, and plant growth regulators, as well as various inorganic ions such as phosphorus, magnesium, potassium, and sodium^53^,^54^. The inhibitory effect of a high BH concentration on seedling growth may be caused by excess carbohydrates or other nutrients in the medium which can prevent plantlet growth *in vitro*^6^.

A high survival of *in vitro Paphiopedilum* plantlets was possible when they were transplanted to Chilean sphagnum moss^5^. However, the roots of the transplanted plantlets elongated slowly and few new roots formed. Therefore, Chilean sphagnum moss is an appropriate substrate only for temporary planting of *Paphiopedilum* plantlets derived from *in vitro*. Two to three months after transplanting, plantlets must be transplanted once again to another substrate. In this study, Zhijing stone for orchids was the most suitable substrate, together with two other substrate mixtures 1 and 3, which contain Zhijing stone for orchids, while commercial sand for orchids was least effective. This may be because Zhijing stone for orchids has good water holding capacity and water permeability, commercial sand for orchids has good water permeability and bad water holding capacity, while Chilean sphagnum moss has good water holding capacity and bad water permeability (data not shown).

In conclusion, the present study documents, for the first time and in considerable detail, the degree to which seed maturity of *P. armeniacum* is a critical factor for seed germination and subsequent seedling development *in vitro* and analyzes the relationships between embryo morphology, seed germination and TTC staining, factors that have to date not yet been studied in this genus. The optimum procedure for seed germination and subsequent development of *P. armeniacum* is to sow 95 to 110 DAP seeds aseptically on ⅛MS or ¼MS media. Mature dry seeds need to be pretreated with 1.0% NaOCl solution for 90 min or with 40 kHz of ultrasound for 8 min to enhance seed germination. This procedure might also serve as a useful method to improve seed germination in other terrestrial orchid genera.

## Materials and Methods

### Plant material

About 1000 *P. armeniacum* S. C. Chen et F. Y. Liu plants were maintained in a glass greenhouse with a water curtain and blowers for cooling and ventilation in the South China Botanical Garden, Guangzhou, China. The plants were potted in a substrate of Zhijing stone for orchids (Northridge Enterprise Co. Ltd., Taiwan) under no more than 800 μmol m^−2^ s^−1^ natural light maintained by a sunshade net. The key characteristics of Zhijing stone for orchids are: pH 5.7, EC 88 μs cm^−1^, unit weight 0.89 g cm^−3^, total porosity 69.2% and water-holding porosity 32.6%. The average temperature and relative humidity ranged from 10–32 °C and 70–98%, respectively. Initial trials indicated that the setting percentage and seed viability from self-pollination were not significantly different to cross-pollination: setting percentage exceeded 90% in both cases. Therefore, the flowers from three-year-old adult plants were labeled and artificially self-pollinated by transferring pollen onto the stigma of the same flower as they became fully opened in March to April. For the experiments, 800 capsules were obtained and approximately 100,000 seeds/capsule.

### Histological and histochemical studies

Capsules of different developmental stages from 38 to 122 DAP were collected at 7-day intervals. Capsules are sliced horizontally and fixed in a solution of FAA (50% ethanol: acetic acid: formalin; 89:6:5, v/v) at 4 °C overnight. After fixation, samples were rinsed six times within 2 h with distilled water, then dehydrated in a graded ethanol series: 30% ethanol for 20 min, 50% ethanol for 20 min, 70% ethanol at 4 °C overnight, 80% ethanol for 20 min, 90% ethanol for 20 min, 100% ethanol for 30 min while the last step was repeated once more. After dehydration, the samples were immersed in epoxy propane for 30 min then re-immersed for the same time period. Samples were transferred to Spurr’s resin for 6 h at room temperature then immersed in fresh Spurr’s resin at 4 °C overnight. Finally, the materials were embedded in Spurr’s resin and baked at 70 °C for 48 h. Semi-thin sections (2 μm thick) were cut with glass knives on a LKB-11800 microtome (LKB Ltd., Uppsala, Sweden). The sections were stained with 1 mg ml^−1^ toluidine blue (Sigma Chemical Co., St. Louis, MO, USA) for 10 min, then washed in distilled water and mounted in water containing 0.1% *n*-propyl gallate (Sigma Chemical Co.), an antifading compound, as detailed by O’Brien and McCully^55^. The fluorescence pattern of toluidine blue was examined using an epifluorescence microscope (Axioskop 2, Carl Zeiss AG, Oberkochen, Germany) equipped with a Zeiss filter set 15 (546/12 nm excitation filter and 590 emission barrier filter)^56^. The sections were observed and images were captured digitally using a CCD camera attached to a light microscope (Axioskop 2) to observe the microscopic structural features of seeds, including starch and lipid globules.

Mature seed morphology (Fig. 3a) was observed by scanning electron microscopy (SEM) using the method reported in Zeng *et al.*^5^ Samples were fixed in 3% (v/v) glutaraldehyde in 0.1 phosphate buffer (pH 7.0) for 12 h and dehydrated in an ethanol series [30%–50%–70%–80%–90%–100% (v/v) in water for 10 min each step], followed by treatment three times with *tert*-butanol, for 10 min each time. The samples were dried with a JFD-310 freeze dryer, affixed to aluminium stubs and coated with gold palladium using a JFC-1600 Fine Coater. The samples were examined with a JSM-6360LV scanning electron microscope (JEOL, Tokyo, Japan).

### Effect of the degree of seed maturity on TTC staining and *in vitro* germination

To determine the influence of the degree of seed maturity on TTC staining and seed germination, 10 seed capsules were collected at different developmental stages – every 10 days from 45 to 180 DAP, except for a 15-day interval from 95 to 110 DAP during which laboratory staff was limited. A similar experimental design had been used by Zeng *et al.*^5^ Seed capsules were surface sterilized by dipping into 75% (v/v) ethanol for 2 min, followed by agitation for 15 min in 1 g l^−1^ mercuric chloride (HgCl_2_) and 0.05% (w/v) Tween 20, after which the capsules were rinsed four times with sterile distilled water (SDW). TTC staining used *ca*. 300 seeds from each collection date that were soaked in filtered TTC solution (1 g in 100 ml phosphate buffer, pH 7.0) for 24 h in the dark at 25 °C and rinsed five times in SDW. Seeds were viewed using a Leica S8APO microscope (Wetzlar, Germany). Embryos that were completely colored pink to red were considered to be viable while seeds that were partially colored, white, yellow or brown were assumed to not be viable.

Based on initial trials, quarter-strength MS medium (one quarter of macro- and micronutrients; ¼MS) medium^23^ supplemented with 0.5 mg l^−1^ NAA, 10% CW (v/v) and 1.0 g l^−1^ activated charcoal (AC) was suitable for seed germination. Therefore, at each collection date, surface-disinfected capsules were cut open longitudinally, seeds were scooped out with sterile forceps and placed on this medium. For each treatment, *ca*. 300 seeds from five capsules were cultured in a 500-ml culture flask containing 90 ml of medium. All experiments consisted of three independent replicates with 10 culture flasks per replicate. Cultures were observed every 30 days for signs of germination and subsequent protocorm development using a Leica S8APO microscope. Developmental stages (Fig. 3b–g) were adapted from Zeng *et al.*^6^, as follows: “(1) Stage 0, ungerminated seed with embryo and not rupturing the testa; (2) stage 1, rupture of the testa by enlarging embryo; (3) stage 2, appearance of the shoot (=protomeristem) and/or rhizoids; (4) stage 3, appearance of the shoot and rhizoids; (5) stage 4, emergence and elongation of first leaf or more roots present; (6) stage 5 presence of two or more leaves and roots (=seedling).” The percentage of seed/protocorms/seedlings at each developmental stage was calculated by dividing the number of seed/protocorms/seedlings in each stage by the total number of cultured seeds in each flask ×100 in each flask at 120 days and 180 days. In this equation, seeds included seeds with and without an embryo. Germination was considered to have occurred only if a swollen embryo was present and if the testa had ruptured (Stage 1).

### Effect of basal media on germination *in vitro*

To study the effect of inorganic salts on seed germination and subsequent protocorm development at different collection times, 95 and 110 DAP seeds from surface-disinfected capsules were placed onto 10 basal sowing media: (1) MS^23^, (2) half-strength MS (½ MS macro- and micronutrients), (3) ¼MS, (4) ⅛MS, (5) Knudson’s C (KC)^57^, (6) Vacin and Went (VW)^58^, (7) Robert Ernst (RE)^59^, (8) Thomale GD^60^, (9) Hyponex N026^52^, (10) Hyponex N016^5^. All basal media were supplemented with 1.0 g l^−1^ AC, 20 g l^−1^ sucrose, 10% CW, 0.5 mg l^−1^ NAA and 5.2 g l^−1^ agar (Huankai Microbial Sci. & Tech, Co., Ltd., Guangzhou, China).

### Effect of NaOCl treatment on TTC staining and germination of maturity seeds

To evaluate the effect of NaOCl on seed germination percentage, mature seeds (180 DAP) dried on silica gel in a sealed glass jar showing highest levels of germination were soaked in 1 g l^−1^ HgCl_2_ for 10 min (control), then in NaOCl containing 0.5%, 1.0% or 1.5% available chlorine for 30, 60, 90 and 120 min. After these pretreatments, seeds were rinsed three times with sterilized water (filtered through sterilized filter paper; Hangzhou WoHua Filter Paper Co., Ltd., Hangzhou, China) and then placed on ¼MS medium containing 10% CW, 0.5 mg l^−1^ NAA and 1.0 g l^−1^ AC or stained by TTC and viewed using a Leica DFC 450 stereomicroscope. For each treatment, *ca*. 300 seeds were cultured in a 500-ml culture flask containing 90 ml of medium. All experiments consisted of three independent replicates with 10 culture flasks per replicate.

### Effect of ultrasonication pretreatment on TTC staining and germination of maturity seeds

The sterilized capsules were split with a sterilized scalpel after disinfection and mature seed (180 DAP) dried on silica gel in a sealed glass jar were placed into a 50-ml aseptic conical tube. Seeds were suspended by adding 10 ml of SDW and ultrasonicated with an Ultrasonic Cleaning Machine (SB-5200 DTN, Scientz, Ningbo, China) at 40 kHz for 2, 4, 6, 8, or 10 min at room temperature. Ultrasonicated seeds were stained by TTC or transferred onto ¼MS medium as described above. For each treatment, *ca*. 300 seeds were cultured in a 500-ml culture flask containing 90 ml of medium. All experiments consisted of three independent replicates with 10 culture flasks per replicate.

### Effect of organic amendments on plantlets growth *in vitro*

Following initial trials, ¼MS medium supplemented with 1.0 mg l^−1^ NAA, 1.0 g l^−1^ peptone, and 1.0 g l^−1^ AC was found to be suitable for the growth of plantlets shorter than 2 cm in the first subculture while Hyponex N026 medium supplemented with 1.0 mg l^−1^ NAA, 1.0 g l^−1^ peptone, 1.0 g l^−1^ AC and appropriate organic amendments were beneficial for the growth of plantlets about 2-cm in height with 2–3 leaves and 2–3 roots. The status of plantlet growth (mean number of shoots per seedling, height of tallest shoot, number of leaves of tallest shoot, number of roots, length and width of longest root) were assessed on plantlets growing on Hyponex N026 media containing 50, 100, 150 g l^−1^ CW, CH, PH, or 25, 50, 100 g l^−1^ BH). The CW used in these experiments was obtained from 6- to 7-month-old green coconuts from Hainan province, China and was filtered through one sheet of filter paper. The fruits for the three organic amendments (CH, PH and BH) were purchased from a local supermarket and the homogenates were obtained after peeling, then homogenizing. Fruits purchased for the three organic amendments from different commercial sources had no significant influence on the efficiency of the culture medium. All experiments consisted of three independent replicates with 10 culture flasks per replicate, with 20 plantlets in each flask.

### Greenhouse acclimatization

*In vitro* propagated plantlets 5-cm in height or taller were transferred to natural conditions for acclimation for 7 days, then transplanted into pots with Chilean sphagnum moss, sieved peat, Zhijing stone for orchids or mixed medium 1 [Zhijing stone for orchids: sieved peat: shattered fir bark; 2: 1: 1 (v/v)], mixed medium 2 [commercial sand for orchids: sieved peat: shattered fir bark; 2: 1: 1 (v/v)], or mixed medium 3 [Zhijing stone for orchids: coconut bran: shattered fir bark; 2: 1: 1 (v/v)] between April and May. The transplanted plantlets were grown in a greenhouse with a water curtain and blowers for cooling and ventilation under no more than 800 μmol m^−2^ s^−1^ natural light with sunshade nets (Zhejiang Luqiao Fangyuan Mesh Factory, Taizhou, China). Plantlets were watered at 1–2-day intervals. Average temperatures ranged from 20 to 32 °C and humidity levels ranged from 70 to 98%. The percentage of plantlet survival was recorded 90 and 180 days after transplanting. Each experiment consisted of three independent replicates with 100 plantlets per replicate.

### Culture conditions

Whenever no special illumination requirements existed, then all cultures were incubated in 500-ml conical flasks closed with perforated rubber stoppers and plugged with cotton. Each flask contained 90 ml of medium that in turn contained 20 g l^−1^ sucrose and 5.2 g l^−1^ agar. Medium pH was adjusted to 5.5 with 56.11 g l^−1^ (1 mol l^–1^ KOH) and 36.46 g l^−1^ (1 mol l^–1^) HCl before autoclaving at 121 °C for 18 min at 1.06 kg cm^–2^. The CW used in these experiments was the same as that obtained above. The cultures were incubated at 25 ± 1 °C with a 16-h photoperiod under cool white fluorescent lamps delivering a photosynthetic photon flux density of *ca*. 45 μmol m^–2^ s^–1^.

### Data analysis

All experiments were established in a completely randomized design. The data were analyzed with SPSS 17.0 for Windows (Microsoft Corp., Washington, USA) and expressed as means ± standard error (SE) using one-way analysis of variance (ANOVA) followed by Duncan’s multiple range test (DMRT) at *P* = 0.05. Correlations were determined as Pearson’s correlation coefficient at *P* = 0.05 or *P* = 0.01.

## Additional Information

**How to cite this article**: Zhang, Y.-Y. *et al.* Embryo development in association with asymbiotic seed germination *in vitro* of *Paphiopedilum armeniacum* S. C. Chen et F. Y. Liu. *Sci. Rep.* **5**, 16356; doi: 10.1038/srep16356 (2015).

## Figures and Tables

**Figure 1:**
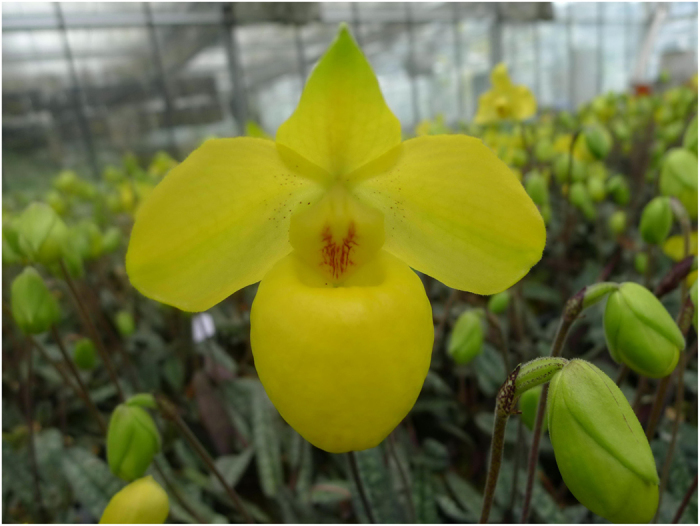
Flowering plants of *Paphiopedilum armeniacum* S. C. Chen et F. Y. Liu were maintained in a greenhouse with a water curtain and blowers in the South China Botanical Garden, Guangzhou, China (Photographed by Songjun Zeng).

**Table 1:** Major microscopic structural events taking place in developing capsules of *Paphiopedilum armeniacum* after pollination.

Days after pollination	Developmental stage	Seed color
31	Ovule development	—
38	Fertilization	—
45	Zygote	White
52	Two-cell pre-embryo	White
59	Three-cell pre-embryo	Yellowish white
66	T-shaped pre-embryo with four cells	Yellowish white
73	Six-cell pre-embryo	A mixture of yellow and light brown
80	Multi-cell early globular embryo with curving suspensor	A mixture of yellow and light brown
87	Preliminary globular embryo formation; the outside-layer cells of the outer integument begin to dehydrate and the suspensor starts to degenerate	Light brown
94	Globular embryo formation; the inner-layer cells of the outer integument begin to dehydrate as the suspensor degenerates; starch and lipid globules accumulate as nutritional storage	Light brown
101	Globular embryo development; the suspensor degenerates and there is additional starch and lipid globule accumulation	Brown
108	The inner testa disappears, and the number of starch and lipid globules increases further	Brown
115	Globular embryo is very close to a testa rich with starch and lipid globules	Brown
122	The embryo is fully mature with a compact testa and no further morphological changes	Dark
129	Mature seeds	Dark
143	Seed desiccation	Dark
150–213	Dry and mature seeds	Dark
220	Ripe capsule that splits	Dark

**Table 2:** Effect of the degree of seed maturity on *in vitro* germination of *Paphiopedilum armeniacum* seeds on quarter-strength MS (macro- and micronutrients) medium supplemented with 0.5 mg l^−1^ NAA, 10% (v/v) CW and 1.0 g l^−1^ AC after culture for 120 days.

Days after pollination	Seedlings in each developmental stage (%)						Total germination (stages 1–5) (%)	Seed TTC staining (%)
	Stage* 0	Stage 1	Stage 2	Stage 3	Stage 4	Stage 5		
45	100a	0k	0e	0d	0b	0	0j	0j
55	95.8 ± 0.2b	4.2 ± 0.2j	0e	0d	0b	0	4.2 ± 0.2iA	4.8 ± 0.4ghA
65	94.9 ± 0.3bc	5.1 ± 0.3ij	0e	0d	0b	0	5.1 ± 0.3hiA	6.7 ± 0.4fgA
75	82.2 ± 0.4e	17.8 ± 0.4f	0e	0d	0b	0	17.8 ± 0.4fB	22.3 ± 1.5dA
85	56.3 ± 1.1h	43.7 ± 1.1c	0e	0d	0b	0	43.7 ± 1.1cB	52.3 ± 1.5cA
95	3.1 ± 0.2j	96.0 ± 0.6a	0.9 ± 0.1b	0d	0b	0	96.9 ± 0.7aA	95.7 ± 0.7aA
110	33.1 ± 1.0i	65.8 ± 1.0b	1.1 ± 0.1a	0d	0b	0	66.9 ± 1.0bB	82.3 ± 1.5bA
120	62.5 ± 1.5g	36.7 ± 1.4d	0.5 ± 0.1c	0.3 ± 0.0a	0b	0	37.5 ± 1.5dA	20.0 ± 1.2dB
130	69.9 ± 0.6f	29.7 ± 0.6e	0.4 ± 0.0d	0.1 ± 0.0c	0b	0	30.2 ± 0.6eA	15.3 ± 1.5eB
140	83.9 ± 1.6e	14.5 ± 2.8g	1.0 ± 0.1a	0.2 ± 0.0b	0.5 ± 0.0a	0	16.1 ± 1.6fA	8.8 ± 0.6fB
150	90.3 ± 0.7d	9.7 ± 0.7h	0e	0d	0b	0	9.7 ± 0.7gA	5.2 ± 0.4ghB
160	93.5 ± 0.1bc	7.2 ± 0.4hi	0e	0d	0b	0	7.2 ± 0.4ghA	3.2 ± 0.2hiB
170	92.7 ± 0.7cd	7.3 ± 0.7hi	0e	0d	0b	0	7.3 ± 0.7g hA	2.7 ± 0.3hijB
180	95.5 ± 0.4b	4.5 ± 0.4j	0e	0d	0b	0	4.5 ± 0.4iA	1.3 ± 0.3ijB

For each treatment, approximately 300 seeds were cultured in a 500-ml culture flask containing 90 ml of medium. All experiments consisted of three independent replicates with 10 culture flasks per replicate. Values followed by different lower-case letters within a column or by different capital letters within a row are significantly different at *P* < 0.05 according to DMRT. Each mean is based on microscopic observations. * See Zeng *et al.*^6^ for detailed explanation of stages 0–5. AC, activated charcoal; CW, coconut water; NAA, α-naphthaleneacetic acid.

**Table 3:** Effect of the degree of seed maturity on *in vitro* germination of *Paphiopedilum armeniacum* seeds on quarter-strength MS (macro- and micronutrients) medium supplemented with 0.5 mg l^−1^ NAA, 10% (v/v) CW and 1.0 g l^−1^ AC after culture for 180 days.

Days after pollination	Seedlings in each developmental stage (%)							Total germination (stages 1–5) (%)
	Stage* 0	Stage 1	Stage 2	Stage 3	Stage 4	Stage 5	Protocorm necrosis	
45	100a	0d	0d	0e	0e	0f	0h	0j
55	94.0 ± 0.3b	0d	0d	0e	0e	0f	6.0 ± 0.3cd	6.5 ± 0.3i
65	90.8 ± 1.0c	2.2 ± 0.2c	0d	0e	0e	0f	7.0 ± 0.9bc	9.2 ± 1.0h
75	79.2 ± 0.4f	3.1 ± 0.2b	2.2 ± 0.2c	2.0 ± 0.1d	2.8 ± 0.2c	5.2 ± 0.1g	5.5 ± 0.1d	20.8 ± 0.4e
85	60.3 ± 0.5g	3.0 ± 0.1b	2.0 ± 0.1c	1.9 ± 0.1d	10.4 ± 0.8a	14.0 ± 1.2d	7.7 ± 0.2b	39.7 ± 0.5d
95	3.1 ± 0.5j	15.0 ± 0.5a	10.4 ± 1.1a	10.7 ± 0.9a	10.0 ± 1.2a	30.7 ± 1.2b	20.0 ± 1.2a	96.2 ± 1.0a
110	25.0 ± 2.6i	2.1 ± 0.1c	3.0 ± 0.3b	5.0 ± 0.2b	10.0 ± 0.3a	50.0 ± 2.9a	4.8 ± 0.4de	75.0 ± 2.6b
120	55.5 ± 0.8h	0d	1.8 ± 0.2c	2.9 ± 0.2c	4.9 ± 0.5b	30.0 ± 1.2b	4.9 ± 0.3de	44.5 ± 0.8c
130	60.3 ± 0.2g	0d	0d	5.1 ± 0.3b	4.8 ± 0.2b	26.3 ± 0.2c	3.0 ± 0.1fg	39.7 ± 0.2d
140	81.8 ± 0.7e	0d	0d	1.7 ± 0.2d	1.7 ± 0.1cd	12.2 ± 0.4de	2.7 ± 0.2fg	18.2 ± 0.7ef
150	84.3 ± 0.3de	0d	0d	0e	1.1 ± 0.2de	10.7 ± 0.3ef	3.9 ± 0.4ef	15.7 ± 0.4fg
160	86.6 ± 1.2d	0d	0d	0e	1.3 ± 0.2de	8.0 ± 0.9fg	4.0 ± 0.1ef	13.3 ± 1.2g
170	90.3 ± 0.2c	0d	0d	0e	2.0 ± 0.2cd	5.0 ± 0.2g	1.8 ± 0.2g	9.7 ± 0.2h
180	92.7 ± 0.3bc	0d	0d	0e	2.0 ± 0.1cd	5.3 ± 0.2g	0h	7.3 ± 0.3hi

For each treatment, approximately 300 seeds were cultured in a 500-ml culture flask containing 90 ml of medium. All experiments consisted of three independent replicates with 10 culture flasks per replicate. Values followed by different lower-case letters within a column are significantly different at *P* < 0.05 according to DMRT. Each mean is based on microscopic observations. * See Zeng *et al.*^6^ for detailed explanation of stages 0–5. AC, activated charcoal; CW, coconut water; NAA, α-naphthaleneacetic acid.

**Table 4:** Effect of basal medium supplemented with 0.5 mg l^−1^ NAA, 10% (v/v) CW and 1.0 g l^−1^ AC on germination and development of 95 DAP *Paphiopedilum armeniacum* seeds cultured for 180 days.

Basal medium	Seedlings in each developmental stage (%)							Total germination (stages 1–5) (%)
	Stage* 0	Stage 1	Stage 2	Stage 3	Stage 4	Stage 5	Protocorm necrosis	
MS	62.6 ± 0.6a	1.7 ± 0.3e	4.7 ± 0.4bc	7.1 ± 0.4de	5.2 ± 0.6fg	4.8 ± 0.4h	14.0 ± 0.6cd	37.4 ± 0.6g
⅛MS	42.3 ± 0.8d	4.1 ± 0.5c	3.5 ± 0.4c	5.8 ± 0.4ef	12.7 ± 0.6c	20.0 ± 1.2e	11.7 ± 0.9de	57.7 ± 1.4d
¼MS	3.8 ± 1.0f	15.0 ± 0.5a	10.4 ± 1.1a	10.7 ± 0.9a	10.0 ± 1.2d	30.1 ± 1.2c	20.0 ± 1.2b	96.2 ± 1.0a
⅛MS	5.7 ± 1.1f	4.1 ± 0.2c	5.9 ± 0.4b	9.7 ± 0.4ab	20.0 ± 1.0a	40.5 ± 1.4a	14.2 ± 0.4cd	94.3 ± 1.1a
KC	55.8 ± 0.5b	1.3 ± 0.3e	4.0 ± 0.6c	5.3 ± 0.2f	7.9 ± 0.4e	15.0 ± 0.8f	10.7 ± 1.2ef	44.2 ± 0.5f
VW	46.8 ± 1.0c	7.8 ± 0.7b	4.7 ± 0.2bc	7.5 ± 0.3d	6.4 ± 0.3efg	4.1 ± 0.6h	22.7 ± 1.5a	53.2 ± 1.0e
RE	34.8 ± 1.4e	2.5 ± 0.3de	3.7 ± 0.2c	8.3 ± 0.4bcd	10.8 ± 0.6cd	24.5 ± 2.3d	15.3 ± 0.9c	65.2 ± 1.4c
Thomale GD	49.6 ± 1.4c	2.3 ± 0.4de	4.7 ± 0.4bc	9.3 ± 0.7abc	4.8 ± 0.2g	21.3 ± 0.9e	8.0 ± 0.6f	50.4 ± 1.4e
Hyponex N026	34.8 ± 1.0e	2.7 ± 0.4de	4.3 ± 0.2c	7.8 ± 0.4cd	16.4 ± .7b	35.4 ± 0.8b	8.5 ± 0.5f	75.2 ± 1.0b
Hyponex N016	62.4 ± 1.2a	3.3 ± 0.3cd	4.3 ± 0.3bc	5.2 ± 0.4f	7.1 ± 0.4ef	8.9 ± 0.6g	8.7 ± 0.3f	37.6 ± 1.2g

For each treatment, approximately 300 seeds were cultured in a 500-ml culture flask containing 90 ml of medium. All experiments consisted of three independent replicates with 10 culture flasks per replicate. Values followed by different lower-case letters within a column are significantly different at *P* < 0.05 according to DMRT. Each mean is based on microscopic observations. AC, activated charcoal; CW, coconut water; KC, Knudson’s C medium (Knudson^57^); MS, Murashige and Skoog medium (Murashige and Skoog^23^); NAA, α-naphthaleneacetic acid; RE, Robert Ernst medium (Arditti^59^); VW, Vacin and Went medium (Vacin and Went^58^). *See Zeng *et al.*^6^ for detailed explanation of stages 0–5.

**Table 5:** Effect of basal medium supplemented with 0.5 mg l^−1^ NAA, 10% (v/v) CW and 1.0 g l^−1^ AC on germination and development of 110 DAP *Paphiopedilum armeniacum* seeds cultured for 180 days.

Basal medium	Seedlings in each developmental stage (%)							Total germination (stages 1–5) (%)
	Stage* 0	Stage 1	Stage 2	Stage 3	Stage 4	Stage 5	Protocorm necrosis (%)	
MS	54.5 ± 0.7a	1.17 ± 0.2e	1.9 ± 0.2d	2.90 ± 0.2d	4.7 ± 0.4f	9.8 ± 1.0e	25.0 ± 1.2b	45.5 ± 0.7f
⅛MS	34.3 ± 1.4d	2.83 ± 0.4bc	4.1 ± 0.3b	7.33 ± 0.4b	14.5 ± 0.9ab	28.5 ± 2.2c	8.5 ± 0.9ef	65.7 ± 1.4c
¼MS	25.0 ± 2.6f	2.10 ± 0.1cde	3.0 ± 0.3c	5.03 ± 0.2c	10.0 ± 0.3d	50.0 ± 2.9a	4.8 ± 0.4g	75.0 ± 2.6a
⅛MS	30.8 ± 0.8de	3.83 ± 0.6b	4.3 ± 0.4ab	4.67 ± 0.3c	15.2 ± 1.0a	32.7 ± 1.2c	8.5 ± 0.8ef	69.2 ± 0.8bc
KC	50.3 ± 1.1b	2.27 ± 1.5cd	2.7 ± 0.2cd	4.50 ± 0.3c	7.2 ± 0.9e	22.8 ± 1.5d	10.2 ± 1.0de	49.7 ± 1.1e
VW	34.9 ± 1.9d	5.07 ± 0.3a	4.6 ± 0.4ab	10.20 ± 0.5a	10.2 ± 1.0d	4.8 ± 0.4f	30.3 ± 1.2a	65.1 ± 1.9c
RE	27.4 ± 0.4ef	3.00 ± 0.1bc	2.3 ± 0.3cd	4.93 ± 0.2c	12.2 ± 0.4bcd	29.2 ± 1.9c	21.0 ± 1.0c	72.6 ± 0.4ab
Thomale GD	41.7 ± 0.9c	4.83 ± 0.4a	2.7 ± 0.2cd	7.50 ± 0.3b	11.7 ± 0.9cd	20.0 ± 1.2d	11.7 ± 0.9d	58.3 ± 0.9d
Hyponex N026	31.6 ± 0.9d	2.50 ± 0.3cd	3.0 ± 0.3c	4.90 ± 0.4c	13.0 ± 1.2abc	38.0 ± 1.2b	7.0 ± 0.6fg	68.4 ± 0.9c
Hyponex N016	55.4 ± 0.5a	1.67 ± 0.3d	5.2 ± 0.2a	4.83 ± 0.6c	10.9 ± 0.6cd	11.3 ± 0.7e	10.7 ± 1.3de	44.6 ± 0.5f

For each treatment, approximately 300 seeds were cultured in a 500-ml culture flask containing 90 ml of medium. All experiments consisted of three independent replicates with 10 culture flasks per replicate. Values followed by different lower-case letters within a column are significantly different at *P* < 0.05 according to DMRT. Each mean is based on microscopic observations. AC, activated charcoal; CW, coconut water; KC, Knudson’s C medium (Knudson^57^); MS, Murashige and Skoog medium (Murashige and Skoog^23^); NAA, α-naphthaleneacetic acid; RE, Robert Ernst medium (Arditti^59^); VW, Vacin and Went medium (Vacin and Went^58^). * See Zeng *et al.*^6^ for detailed explanation of stages 0–5.

**Table 6:** Effects of NaClO pretreatments on germination of mature seeds (180 DAP) of *Paphiopedilum armeniacum* on quarter-strength MS medium supplemented with 0.5 mg l^−1^ NAA, 10% (v/v) CW and 1.0 g l^−1^ AC after culture for 180 days.

Available chlorine (%)	Treatment duration (min)	Seedlings in each developmental stage (%)							
		Stage** 0	Stage 1	Stage 2	Stage 3	Stage 4	Stage 5	Total germination (stages 1–5)	Seed TTC staining (%)
Control*	0	100a	0f	0h	0e	0g	0f	0h	0h
0.5	30	94.3 ± 0.6b	2.9 ± 0.2b	2.0 ± 0.6ef	0.8 ± 0.2d	0g	0f	5.7 ± 0.6g	2.5 ± 0.4g
0.5	60	90.2 ± 0.3c	3.0 ± 0.3b	2.7 ± 0.4cde	2.0 ± 0.3c	2.2 ± 0.1f	0f	9.8 ± 0.3f	7.7 ± 0.4f
0.5	90	83.2 ± 0.8ef	1.3 ± 0.3cde	3.8 ± 0.4ab	2.9 ± 0.2b	4.7 ± 0.6cd	4.1 ± 0.2d	16.8 ± 0.8cd	14.3 ± 0.3d
0.5	120	79.6 ± 0.8g	0f	4.0 ± 0.6a	3.2 ± 0.3ab	6.1 ± 0.6b	7.1 ± 0.4b	20.4 ± 0.8b	23.4 ± 1.0b
1.0	30	87.2 ± 0.5d	3.9 ± 0.2a	3.3 ± 0.2abc	2.7 ± 0.4bc	2.8 ± 0.3ef	0f	12.8 ± 0.5e	7.8 ± 0.4f
1.0	60	83.7 ± 0.4e	1.8 ± 0.2c	3.2 ± 0.1abcd	2.7 ± 0.3bc	3.5 ± 0.4de	5.1 ± 0.4c	16.3 ± 0.4d	20.0 ± 1.2c
1.0	90	84.6 ± 0.58e	0.6 ± 0.2e	2.2 ± 0.2def	3.3 ± 0.2ab	8.5 ± 0.8a	10.5 ± 0.5a	25.4 ± 0.6a	29.0 ± 2.22
1.0	120	81.7 ± 0.8f	1.5 ± 0.3cd	0.8 ± 0.2gh	3.9 ± 0.2a	5.0 ± 0.3bc	7.0 ± 0.4b	18.3 ± 0.8b	19.7 ± 0.9c
1.5	30	84.6 ± 0.4e	3.3 ± 0.3ab	2.6 ± 0.3cde	3.2 ± 0.2ab	2.0 ± 0.3f	4.2 ± 0.2d	15.4 ± 0.4d	11.0 ± 0.6e
1.5	60	81.9 ± 0.5f	1.2 ± 0.2de	3.0 ± 0.1bcd	3.2 ± 0.2ab	3.9 ± 0.2cde	6.9 ± 0.3b	18.1 ± 0.5b	13.0 ± 0.6de
1.5	90	89.7 ± 0.3c	0f	0h	2.6 ± 0.4bc	2.9 ± 0.2ef	4.9 ± 0.3cd	10.3 ± 0.3f	6.8 ± 0.2f
1.5	120	94.5 ± 0.2b	0f	1.6 ± 0.2fg	0.9 ± 0.1d	0.7 ± 0.2g	2.3 ± 0.2e	5.5 ± 0.2g	3.8 ± 0.4g

For each treatment, approximately 300 seeds were cultured in a 500-ml culture flask containing 90 ml of medium. All experiments consisted of three independent replicates with 10 culture flasks per replicate. Values followed by different lower-case letters within a column are significantly different at *P* < 0.05 according to DMRT. *Control: 0.1% HgCl_2_ aqueous solution. ** See Zeng *et al.*^6^ for detailed explanation of stages 0–5. AC, activated charcoal; CW, coconut water; NAA, α-naphthaleneacetic acid.

**Table 7:** Effects of ultrasonic wave pretreatment on germination of mature seeds (180 DAP) of *Paphiopedilum armeniacum* on quarter-strength MS medium supplemented with 0.5 mg l^−1^ NAA, 10% (v/v) CW and 1.0 g l^−1^ AC at 180 days after culture.

Sonication time (min)	Seedlings in each developmental stage (%)							Seed TTC staining (%)
	Stage* 0	Stage 1	Stage 2	Stage 3	Stage 4	Stage 5	Total germination (stages 1–5)	
0	100a	0c	0d	0c	0d	0c	0e	0f
2	93.4 ± 0.5b	4.2 ± 0.4a	2.3 ± 0.4bc	0c	0d	0c	6.6 ± 0.5d	2.7 ± 0.4e
4	87.5 ± 0.4c	2.8 ± 0.2b	3.8 ± 0.4ab	3.2 ± 0.2b	2.6 ± 0.2c	0c	12.5 ± 0.4c	8.7 ± 0.7d
6	80.8 ± 0.9d	2.0 ± 0.3b	3.3 ± 0.2a	4.3 ± 0.6a	3.8 ± 0.3b	5.7 ± 0.1b	19.2 ± 0.9b	15.5 ± 0.8b
8	73.2 ± 1.4e	2.3 ± 0.2b	2.5 ± 0.3bc	4.6 ± 0.2a	5.5 ± 0.3a	10.5 ± 1.6a	25.4 ± 1.6a	19.7 ± 1.5a
10	85.3 ± 0.9c	0c	2.0 ± 0.3c	2.7 ± 0.2b	4.5 ± 0.3b	5.5 ± 0.8b	14.7 ± 0.9c	12.2 ± 1.0c

For each treatment, approximately 300 seeds were cultured in a 500-ml culture flask containing 90 ml of medium. All experiments consisted of three independent replicates with 10 culture flasks per replicate. Values followed by different lower-case letters within a column are significantly different at *P* < 0.05 according to DMRT. Each mean is based on microscopic observations. * See Zeng *et al.*^6^ for detailed explanation of stages 0–5. AC, activated charcoal; CW, coconut water; NAA, α-naphthaleneacetic acid.

**Table 8:** Effects of organic amendments on the *in vitro* growth of 2-cm tall *Paphiopedilum armeniacum* plantlets on Hyponex N026 medium supplemented with 1.0 g l^–1^ peptone, 1.0 mg l^–1^ NAA, 20 g l^–1^ sucrose and 1.0 g l^–1^ AC after culture for 120 days.

Organic amendments (g l^−1^)	Mean number of shoots per seedling	Height of tallest shoot (cm)	Number of leaves of tallest shoot	Number of roots	Length of longest root (cm)	Diameter of longest root (mm)	Growth status of seedlings
Control	1.3 ± 0.0h	5.13 ± 0.1e	4.0 ± 0.1d	3.3 ± 0.2de	4.1 ± 0.1bcde	2.2 ± 0.1g	++
CW 50	2.4 ± 0.1c	5.50 ± 0.1cd	4.3 ± 0.1cd	3.8 ± 0.2bcd	4.2 ± 0.2bcd	2.7 ± 0.1ef	++
CW 100	3.0 ± 0.1b	5.77 ± 0.2bc	4.6 ± 0.1bc	3.0 ± 0.2ef	3.9 ± 0.4de	3.0 ± 0.1cde	+++
CW 150	3.6 ± 0.1a	5.27 ± 0.1de	4.0 ± 0.1d	2.8 ± 0.1f	3.6 ± 0.1e	2.7 ± 0.1ef	++
CH 50	1.5 ± 0.2gh	5.27 ± 0.1de	4.4 ± 0.1cd	3.5 ± 0.2cde	4.4 ± 0.2bcd	2.5 ± 0.1fg	++
CH 100	1.8 ± 0.1efg	5.77 ± 0.1bc	5.0 ± 0.2ab	3.6 ± 0.1cd	4.2 ± 0.2bcd	2.8 ± 0.1ef	++
CH 150	1.6 ± 0.1fgh	5.70 ± 0.1c	4.3 ± 0.1cd	4.3 ± 0.2ab	4.7 ± 0.1ab	3.2 ± 0.1cd	+
PH 50	1.8 ± 0.15efg	5.50 ± 0.1cd	4.3 ± 0.1cd	3.4 ± 0.2de	4.1 ± 0.1cde	3.2 ± 0.1c	++
PH 100	2.1 ± 0.2de	6.03 ± 0.1b	5.0 ± 0.2ab	3.7 ± 0.2cd	4.4 ± 0.2abcd	3.3 ± 0.1c	+++
PH 150	2.0 ± 0.2def	5.67 ± 0.1c	4.9 ± 0.2ab	4.3 ± 0.1ab	4.3 ± 0.1bcd	3.6 ± 0.1b	+
BH 25	2.0 ± 0.1def	5.67 ± 0.0c	4.7 ± 0.2bc	4.0 ± 0.1bc	4.5 ± 0.0abc	2.9 ± 0.0de	++
BH 50	2.2 ± 0.1cd	6.47 ± 0.2a	5.3 ± 0.2a	4.2 ± 0.1ab	4.9 ± 0.1a	3.6 ± 0.1b	+++
BH 100	1.4 ± 0.1h	4.23 ± 0.2f	4.2 ± 0.1cd	4.5 ± 0.2a	4.7 ± 0.2ab	4.6 ± 0.1a	+

For each treatment, 20 seedlings about 2-cm in height with 2–3 leaves and 2-3 roots were cultured in a 500-ml culture flask containing 90 ml of medium. All experiments consisted of three independent replicates with 10 culture flasks per replicate. Means ± SE of 600 replicates with the different letters within columns are significantly different at *P* < 0.05 according to DMRT. +, ++, +++ represents poor, average, and good growth, respectively. AC, activated charcoal; BH, banana homogenate; CH, carrot homogenate; CW, coconut water; NAA, α-naphthaleneacetic acid; PH, potato homogenate.

**Table 9:** Survival rate of *Paphiopedilum armeniacum* seedlings grown on seven supporting substrates at 90 and 180 days after transplanting.

Transplanting substrate	Survival 90 d after transplanting (%)	Survival 180 d after transplanting (%)
Chilean sphagnum moss	90.7 ± 3.0aA	81.0 ± 3.1aB
Sieved peat	79.0 ± 2.1bcA	72.3 ± 1.5bB
Commercial sand for orchids	74.3 ± 2.3cA	63.7 ± 1.9cB
Zhijing stone for orchids	89.3 ± 2.3aA	85.3 ± 1.5aA
Substrate mixture 1*	86.0 ± 3.1abA	81.7 ± 2.0aA
Substrate mixture 2**	79.3 ± 1.8bcA	70.3 ± 1.5bB
Substrate mixture 3***	85.0 ± 1.7abA	79.7 ± 2.0aA

Values followed by different lower-case letters within a column or by different capital letters within a row are significantly different at *P* < 0.05. Each experiment consisted of three independent replicates with 100 plantlets per replicate. * Zhijing stone for orchids: sieved peat: shattered fir bark (2: 1: 1, v/v). ** Commercial sand for orchids: sieved peat: shattered fir bark (2: 1: 1, v/v). *** Zhijing stone for orchids: coconut bran: shattered fir bark (2: 1: 1, v/v).
